# A Microfluidic Flip-Chip Combining Hydrodynamic Trapping and Gravitational Sedimentation for Cell Pairing and Fusion

**DOI:** 10.3390/cells10112855

**Published:** 2021-10-22

**Authors:** Gaurav Pendharkar, Yen-Ta Lu, Chia-Ming Chang, Meng-Ping Lu, Chung-Huan Lu, Chih-Chen Chen, Cheng-Hsien Liu

**Affiliations:** 1 Department of Power Mechanical Engineering, National Tsing Hua University, Hsinchu 30044, Taiwan; g.pendharkar@ieee.org (G.P.); woes6210@gmail.com (C.-H.L.); chihchen@mx.nthu.edu.tw (C.-C.C.); 2 Chest Department, MacKay Memorial Hospital, New Taipei City 10449, Taiwan; ytlhl@mmh.org.tw; 3 Department of Medical Research, MacKay Memorial Hospital, New Taipei City 10449, Taiwan; aming.chang@gmail.com (C.-M.C.); miriam01084@gmail.com (M.-P.L.); 4 Institute of Nanoengineering and Microsystems, National Tsing Hua University, Hsinchu 30044, Taiwan

**Keywords:** hydrodynamic trapping, cell pairing, dielectrophoresis, cell fusion

## Abstract

Cancer cell–immune cell hybrids and cancer immunotherapy have attracted much attention in recent years. The design of efficient cell pairing and fusion chips for hybridoma generation has been, subsequently, a subject of great interest. Here, we report a three-layered integrated Microfluidic Flip-Chip (MFC) consisting of a thin through-hole membrane sandwiched between a mirrored array of microfluidic channels and saw-tooth shaped titanium electrodes on the glass. We discuss the design and operation of MFC and show its applicability for cell fusion. The proposed device combines passive hydrodynamic phenomenon and gravitational sedimentation, which allows the transportation and trapping of homotypic and heterotypic cells in large numbers with pairing efficiencies of 75~78% and fusion efficiencies of 73%. Additionally, we also report properties of fused cells from cell biology perspectives, including combined fluorescence-labeled intracellular materials from THP1 and A549, mixed cell morphology, and cell viability. The MFC can be tuned for pairing and fusion of cells with a similar protocol for different cell types. The MFC can be easily disconnected from the test setup for further analysis.

## 1. Introduction

It has been hypothesized that cell fusion contributes to tumor development and spreading behavior [1]. Therefore, cellular vaccines were produced and described a decade ago, based on the cell fusion of dendritic cells (DCs) and cancer cells to offer hybrid cells sharing a united cytoplasm but keeping the identity of dual nuclei [2,3,4]. Cellular fusion is a process in which two or more cells are merged in an asexual way producing a hybrid cell. The process of hybridoma formation is an essential step for the development of organisms as well as human beings. Extending the use of such hybridomas has led to the development of a tool called cancer immunotherapy [5]. Cancer cell–immune cell hybrids and cancer immunotherapy has attracted much attention in recent years. In addition to cancer immunotherapy [6], applications of cell fusion have long been discussed extensively in a variety of fields such as hybridoma generation [7,8], reprogramming of somatic cells [9], and mammal cloning [10,11]. Cell fusion can be categorized primarily into three types, viz, poly-ethylene glycol (PEG) based fusion (chemical fusion) [12], fusion via viruses (biological) [13], and electroporation (physical) [14].

In vitro techniques for cell fusion have been demonstrated using microfluidic systems. Cell electrofusion in a microfluidic device is a two-step process, cell pairing being the first, followed by cell electroporation. Improving fusion efficiency needs careful design and implementation of the mechanism for bringing the cells together (cell pairing) and the triggering mechanism to initiate membrane fusion. It is necessary to avoid unwanted hybridomas caused by multi-cell fusion or fusion among the same cells [15,16]. Microfluidic-based cell operation has many advantages, such as precise manipulation and high efficiency in cell pairing. There are primarily three cell pairing methods—a chemical method, the use of the electric field, and that of incorporating microstructures. Higher fusion efficiency depends on perfectly paired cells instead of random cell pairing, a common scenario seen in conventional methods. Several microfluidic designs utilizing microchannel geometrical effects, hydrodynamic forces for cell pairing have been demonstrated. The controlled pairing of partner cells has been shown using a high throughput cell pairing and a combination of cell pairing and fusion using chemical conjugation [17], field-free microstructure assisted [18], and electric-field assisted cell pairing with better adaptability using hydrodynamic traps or constriction trapping [19,20,21,22]. Some researchers have demonstrated single cell block printing [23] and accurate one-to-one pairing between the tumor and fibroblasts [24].

Conventional fusion approaches, such as PEG-based fusion, have demonstrated a high probability of fusion among the same cell type, resulting in extensive sample processing and, ultimately, low fusion efficiency. Microfluidic-based cell electrofusion with metal electrode integration has resulted in greater efficiency, lower sample contamination, and increased cell survival. The contraction of the local electric field also minimizes the Joule heating effect and increases cell viability [25]. Several microfluidic designs, such as the use of protruding electrodes [18,21,26,27,28,29,30], and the inclusion of microstructures between electrodes [19,20,31,32,33], helps to modify spatial distribution, which in turn shows electric field enhancement. The electric field constriction ensures the field is concentrated between paired cells. The last step in this process is the electrofusion of cells. In this step, a series of short interval direct current (DC) pulses are applied between the electrodes. The process is followed by applying the alternating current (AC) signal for a short duration to ensure cell–cell contact for complete hybridoma formation. Several new techniques carrying out cell electrofusion have been reported. The electrofusion process inside droplets has been demonstrated by using homogeneous cell types [34]. Hsiao et al. and Yang et al. reported the electrofusion based on optically induced local field enhancement [35,36]. The use of optically induced local field enhancement for electrofusion, although a novel idea, requires expensive instrumentation for the implementation. PEG-based fusion reported by Huang et al. demonstrates centrifugal microfluidics for single-cell trapping [37].

Despite the efforts in improving the cell fusion process, most of the microsystems heavily rely on specialized setups (optical tweezers, high voltage power supply, etc.) as well as on the skills of personnel. Secondly, some of the methods require cellular modification or the use of hypo-osmolar treatment for increasing cell sizes. The current cell fusion microsystems are generally designed for a particular cell size. Integrated realization of flow-through channels for heterogeneous cells types with large variations is another difficulty in optimizing such devices for high efficiency and high throughput. The newly proposed methods offer a limited throughput and cell pairing efficiency (e.g., optical fusion, droplet microfluidics). Several microfluidic chips required pre-treatment of cells and the use of high electric fields, leading to handling difficulties and longer exposure time for cell manipulation.

Here, we propose a Microfluidic Flip-Chip (MFC) using a hydrodynamic approach. The MFC consists of the hook-shaped trapping structures placed in the flow-through channel. The microarrayed trapping structures placed vertically allow sequential loading for cell capture. We have demonstrated cell pairing and fusion using THP1 and A549 cells. The cells are first trapped in hydrodynamic traps and later transferred to fusion wells by flipping the chip. The electrofusion was carried out inside fusion wells with the application of the DC pulse. Then the cells were successfully retrieved from the flip-chip. Finally, the fused cells were successfully retrieved and transferred from the chip to the culture plate. Additionally, we have also analyzed the properties of fused cells from a cell biology perspective, including combined fluorescence-labeled intracellular materials from THP1 and A549, cell morphology, and cell viability.

## 2. Materials and Methods

### 2.1. Device Design

**Figure 1:**
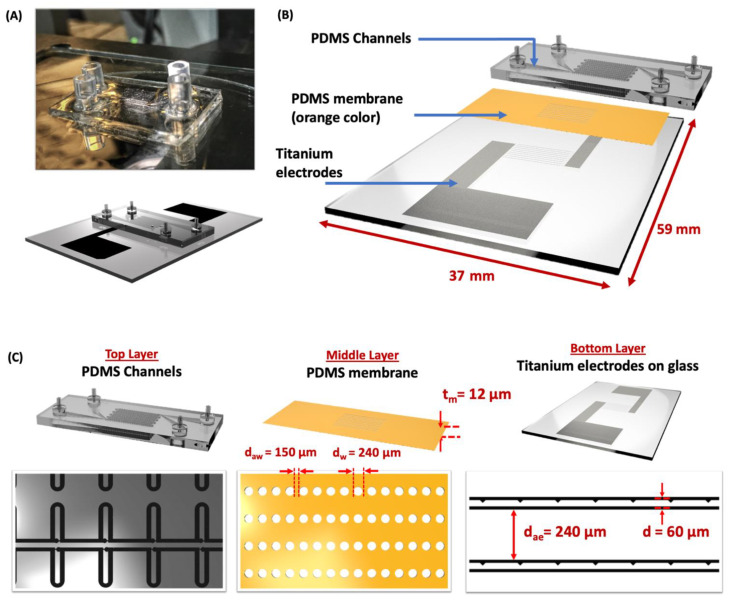
Illustration of Microfluidic Flip-Chip (**A**) Image shows the fabricated MFC using a soft lithography process along with the illustration. (**B**) A graphic illustration of the MFC shows the three-layered structure with PDMS channels as the top layer, through-hole membrane as the middle layer, and titanium electrodes as the third bottom layer. (**C**) The parameters affecting chip performance—the PDMS membrane thickness (t_m_), the diameter of fusion well (d_w_), the distance between adjacent wells (d_aw_), the distance between electrodes (d), and the distance between adjacent electrodes (d_ae_) are as shown. Scale bar: 200 µm.

Figure 1 shows the fabricated MFC using a soft lithography technique [38] (Figure 1A) and a graphic illustration (Figure 1B). The proposed MFC is a three-layered structure consisting of PDMS microchannels as the top layer, a thin PDMS membrane as the middle layer, and titanium electrodes on the glass as the bottom layer (Figure 1C). The MFC consists of 1000 pairs of trapping structures carefully designed by placing them into a straight channel spanned along an area of 59 mm × 37 mm. A narrow hook-shaped channel connects individual trapping sites and is cascaded in series to form an array over a large area. The hook-shaped trapping structures are designed in such a way that only one cell is captured. This array is mirrored vertically to make another set of structures. The two structures operate independently, making it a serial cell loading process. The middle layer, PDMS through-hole membrane, consists of an array of holes called fusion wells with a diameter of d_w_ = 240 µm and distance between adjacent wells d_aw_ = 150 µm arrayed over a large area. The bottom layer consists of saw-tooth-shaped electrodes designed to form a non-uniform electric field, separated by a distance of d = 60 µm. The adjacent electrodes are placed at a distance of d_ae_ = 240 µm. The chip is biased using the AC and DC supply to pair and fuse the cells, respectively.

### 2.2. Device Fabrication

The MFC was fabricated using a soft lithography process. The master mold for microchannels was created using the negative photoresist (SU-8). The SU-8 2015 (MicroChem, Newton, MA, USA) photoresist was spun at 3500 rpm for 30 sec to get the desired feature height of 16~18 µm. The wafer was UV exposed through a photomask for the micro-channel patterns. The development and baking steps were performed as per the manufacturer’s protocol. Following development and baking, the wafer was hard-baked at 150 °C for 30 min. The elastomer base and the curing agent (Sylgard 184, Dow Corning Corporation, Midland, MI, USA) were mixed in the ratio of 10:1, degassed in a vacuum chamber to remove the bubbles inside, to make the applicable PDMS. The devices were then cast by pouring this PDMS on the master mold and cured overnight at 40 °C. The PDMS was peeled off, and individual devices were diced. Holes for fluidic connections were punctured using hole punchers. A 4-inch glass wafer was piranha cleaned, followed by a titanium deposition of 2000 Å using E-Gun evaporation to fabricate electrodes. The wafer was coated with a positive photoresist, exposed under UV light, and further developed for the desired saw-tooth-shaped pattern of electrodes. The unwanted metal was etched using a metal etchant. The middle layer is a thin PDMS membrane (11 ± 2 µm). This is a two-step process. First, SU-8 2015 (SU-8 2015, MicroChem, Newton, MA, USA) photoresist was spun at 2000~2200 rpm for 30 s to get the desired feature height of 20~22 µm. The wafer was UV exposed through a photomask for the micro-channel pattern consisting of an array of pillars. The wafer was developed and baked, followed by a hard bake at 150 °C for 30 min. A thin layer of Teflon was coated on the SU-8 master. In the next step, the PDMS pre-polymer (10:1) was diluted in n-Hexane (Sigma Aldrich, St. Louis, MO, USA) at 1:2 ratios by weight. Dilution with n-hexane decreases the viscosity of PDMS, making the formation of a thin PDMS layer possible. The wafer was then baked at 65 °C for 45 min. Finally, the PDMS layer with micro-channels was bonded on the membrane, and the whole structure was peeled off. Isopropyl alcohol (2-Propanol, Sigma Aldrich, St. Louis, MO, USA) was used as a solvent during the membrane peeling process. This proposed fabrication technique eliminates the handling difficulties making chip reproducibility easier. A pictorial depiction of fabrication steps has been included in the supplementary information (Figure S1).

### 2.3. PDMS Membrane Optimization

We studied two critical processing parameters controlling the thickness of the PDMS membrane, namely, the n-hexane dilution ratio and spinning speed (Figure S5). It should be noted that the thickness of the SU-8 layer consisting of a pillar structure needs to be higher than the required PDMS thickness for the perfect PDMS membrane. We observed an exponential decrease in PDMS thickness with an increase in spin speeds. Another vital parameter affecting PDMS thickness is the n-hexane dilution ratio. Among two different dilution ratios tested (1:1 and 1:2), a steeper slope was observed for a dilution ratio of 1:1.

### 2.4. Modeling and Simulation

Cell damage can occur over time by being exposed to an electric field. Using passive hydrodynamics, MFC allows efficient cell entrapment. Researchers have studied several hydrodynamic-based trapping techniques, including passive approaches, for single-cell studies. One of the earliest and still popular ideas was published by Tan et al. [39], who envisioned employing a dynamic microarray system to capture and then release polystyrene beads. An advantage of systems such as these is that the fluid velocity is not dependent on trapping efficiency. For the trapping structure to work, it is necessary that the trapping structure provides a flow of fluid via the path to keep the volume flow rate above that of the bypass path.

The proposed MFC is a three-layered structure with a through-hole membrane as the middle layer. The mirrored array design with a through-hole membrane in between has not been previously reported. The original design proposed by Tan et al. [39] is independent of the flow rate. However, the presence of the membrane makes fluid velocity a parameter of consideration. At higher flow rates (>3 µL/min), the fluid from one channel flowed across the mirrored channel due to the presence of a membrane. At a lower flow rate (<3 µL/min), we observed a very little cross-channel fluid flow, and hence the leakage effects could be ignored. A fluidic resistance-based model has been designed based on the volumetric flow rate assuming leakage due to the added thin membrane are negligible. Once the dimensions were fixed, we experimentally calculated the exact flow rate needed for the chip to work at its peak efficiency.

**Figure 2:**
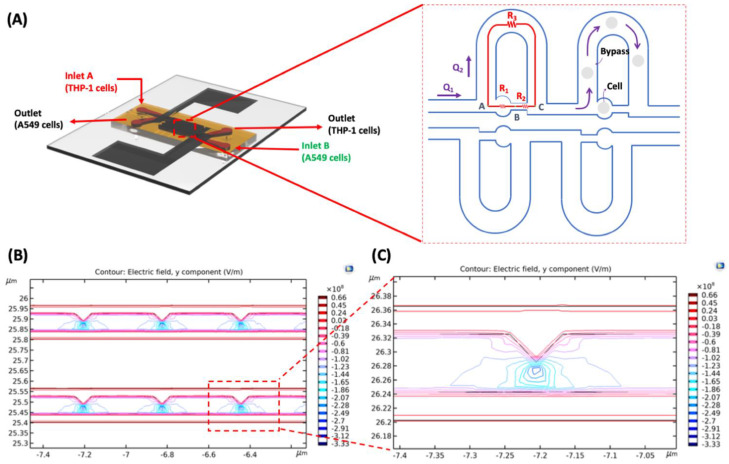
(**A**) Fluidic resistance model for the cell trapping process. The first cell is trapped when there is less flow resistance. After the cell is trapped, the fluidic resistance across this short path is increased, and the cells inside the fluid follow the bypass path. (**B**) FEM-based simulation of saw-tooth shaped electrodes for studying the electric field for maximum fusion efficiencies. (**C**) Zoomed-in view of the simulated electric field near the electrode tip. The cells are attracted to the tip of triangular electrodes for positive-DEP (pDEP).

Figure 2A shows a schematic design of trapping sites that are symmetrical in design. Each trapping structure is made up of a trapping site (path 1: $\bar{ABC}$) with volumetric flow rate Q_1_ and a bypass channel (path 2: $\bar{AC}$) through resistance R_3_ with a volumetric flow rate Q_2_. The flow channels are designed such that the volumetric flow rate Q_1_ in path 1 is higher than the volumetric flow rate Q_2_ in path 2. The majority volume of the medium-containing cells passes through path 1 as the flow resistance is lower than path 2. While flowing through the channels, the individual cells are trapped at the trapping sites, eventually increasing the resistance in path 1. Under this condition, most of the fluid passes through path 2 as the resistance in this path is lower. In this chip, Q_1_/Q_2_ equals 1.35, a critical value for this design (detailed explanation is included in the supplementary material). The decrease in this ratio’s value would not capture the cells; however, a higher value will trap multiple cells. Detailed numerical modeling and functioning are explained in the supplementary material (Figure S2). The MFC uses the dielectrophoresis phenomenon for cell pairing. A neutral particle suspended in a dielectric medium is exposed to a non-uniform electric field. The force exerted on the particle depends primarily on the magnitude and polarity of the charges induced on the particle under the non-uniform electric field. We performed a Finite Element Method (FEM) simulation using COMSOL Multiphysics for the ideal electrode design for our MFC (Figure 2B and Figure S3). The normalized electric field gradient along the path between the two electrodes was analyzed. The electric field lines concentration for the saw-tooth shaped electrode pattern was observed to be more suitable for cell pairing and electrofusion as the highest electric field gradient was observed at the tip of an electrode showing the non-uniform nature of the field. A detailed comparison between the simulation of two electrode patterns has been discussed in the supplementary material. Figure 2C shows the zoom-in view of the y-component of the electric field after applying the AC signal with a frequency of 1 MHz.

### 2.5. Cell Preparation

The A549 (ATCC^®^ CCL185™) is a human lung carcinoma cell line. The A549 cells were cultured in 90% Ham’s F12K medium supplemented with 10% Fetal Bovine Serum (FBS; Invitrogen). The pH of the culture medium containing 2 mM l-glutamine was adjusted to 7.2 by NaOH and HCl. The THP-1 (ATCC^®^ TIB202™) is a human peripheral blood acute monocytic leukemia cell line. The THP-1 cells were maintained in a standard cell culture incubator (5% CO_2_, 95% humidity, 37 °C). Cells were cultured in the Roswell Park Memorial Institute (RPMI) 1640 medium supplemented with 10% Fetal Bovine Serum (FBS), 4.5 g/L glucose, 10 mM HEPES, and 1.0 mM sodium pyruvate, supplemented with 0.05 mM 2-mercaptoethanol. A suspension of THP-1 cells (10^4^ cells/mL) stained in red (CellTracker™ Red CMTPX) and A549 cells (10^4^ cells/mL) stained in green (CellTracker™ Green CMFDA) were used for a better understanding of the pairing and fusion process. Once the operating parameters were optimized, the experiments were repeated using non-labelled cells for long-term cell culture studies.

### 2.6. Experimental Setup

The setup consists of a syringe pump (KDS230, KDScientifc), a function generator (33220A, Agilent Technology, Santa Clara, CA, USA), and a fluorescent microscope (BX51, Olympus, Tokyo, Japan) fitted with a digital microscope camera (SPOT RT3, Diagnostic Instruments, Sterling Heights, MI, USA). The process of cell pairing and fusion was captured using vendor proprietary software SPOT Advanced. The illustration for experimental setup and step-by-step operation has been shown in the supplementary information (Figure S4). The whole process is recorded as a series of images. The images were then analyzed using ImageJ, a public domain image processing program for calculating cell trapping, pairing, and fusion efficiency. After recording the images, the images were converted to the grayscale. After adjusting the threshold, the cells were counted using the analyze particles feature in ImageJ.

### 2.7. Device Operation

#### 2.7.1. Cell Trapping

An essential step before cell loading is cleaning microfluidic devices to avoid bubble formation and bacterial contamination. The MFC was filled with deionized water (DI) and placed in a DI water-filled dish in a desiccator until the air bubbles were removed from the microchannel (approximately 20 min). The chip was later exposed to UV light to sterilize for 30 min. The MFC was conditioned with 1% Bovine Serum Albumin (BSA) and ddH_2_O solution to modify the surface properties of the microchannel as it prevents the adhering of cells to the microchannel wall.

**Figure 3:**
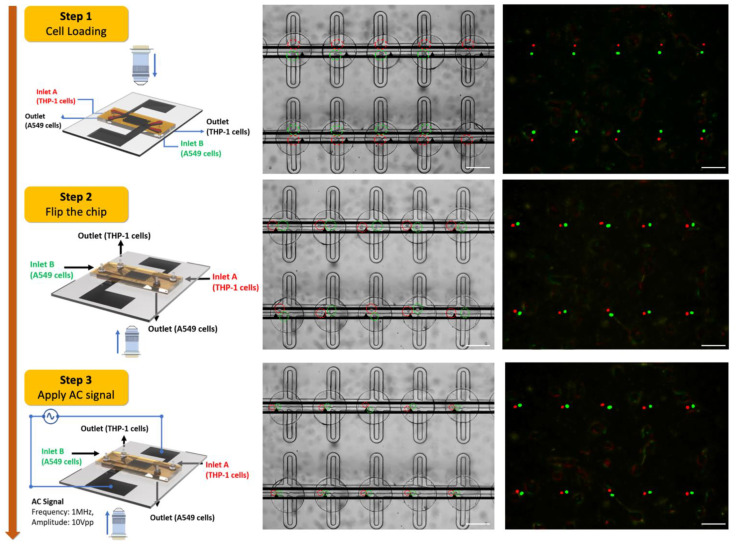
Illustration of MFC device operation for cell loading and pairing. Step 1: THP-1 cells and A549 are loaded from the respective inlets sequentially, followed by a washing step to flush out excess cells. Step 2: The chip is flipped, and the cells are transferred to the fusion wells. Step 3: The cell–cell contact is achieved by applying the AC signal (Frequency: 1MHz, Amplitude: 10V_pp_). The illustration of an objective lens and an arrow indicates the direction from which the image was captured. Scale bar: 200 µm.

The MFC has two inlets and two outlets for the independent loading and unloading operation of cells. The optimal design parameters ensure single-cell trapping and prevent channel clogging throughout the cell loading and unloading process. Figure 3 (Step 1) shows the cell trapping of THP-1 and A549 cells. The cell loading process of THP-1 was accomplished by injecting 1ml of fusion buffer containing cells from Inlet A and collecting the cells from the outlet at a flow rate of 1.5 µL/min using a syringe pump. After most of the trapping sites were filled with cells, a cleaning step was performed. A fusion buffer was flushed into the channels at a reduced flow rate (0.5 µL/min). This process ensured the removal of excess cells while maintaining the already filled trapping sites as intact. Next, the second type of cell, i.e., A549, was introduced from Inlet B with a flow rate of 1.5 µL/min. The cell loading process was continued until most of the trapping sites were filled. A washing step was performed for the removal of excess cells using a fusion buffer. No significant effect concerning cell unloading from the traps was observed during the second step of cell loading.

#### 2.7.2. Cell Pairing and Fusion

After successful cell trapping, the tubing was removed from the chip, and the holes were plugged. The MFC was flipped gently in which the trapping sites are now on the channel’s ceiling, and the fusion wells are on the floor, allowing the captured cells to fall off from the trapping sites to the fusion wells by gravity. After the cells were settled in a fusion well, the uncaptured cells were washed away from the device by slowly injecting a fusion buffer (Figure 3, Step 2). The cell trapping and transferring procedures require 15–18 min approximately. After flipping the chip, all the cells are transferred to the fusion well. An alignment signal (Amplitude: 10V_pp_, Frequency: 1 MHz) was applied between the electrode array. (Figure 3, Step 3). A low conductivity buffer (<200 μS/cm) solution is necessary for the dielectrophoresis phenomenon to occur. It also helps to have better cell viability. The alignment signal induces positive DEP force on the cells aligning them as pairs with high efficiency. In the next step, a DC pulse (Duration: 100 μs, Number of pulses: 10) was applied to induce temporary cell membrane perforation. The optimum value of the DC field helps in cell membrane reconstruction because of the cell’s self-recovering and resealing ability. In addition to this, better cell viability is achieved due to the cytoplasm exchange between paired cells. In the next step, the fused cells are removed by the flowing buffer solution through Outlet A and Inlet B, and the cells are collected from Inlet A and Outlet B.

### 2.8. Image Acquisition and Analysis

The cell pairing and fusion phenomenon can be observed by imaging fluorescence exchange among the cells. The array of images were captured at different times, such as after flipping the chip, after applying the AC signal, and after applying the DC pulse. The application of the DC pulse made the cell membrane unstable and formed reversible membrane pores, leading cells in physical contact to achieve electrofusion.

### 2.9. Statistical Analysis

All experiments were performed in triplicate and the data are presented as mean ± standard deviation (SD). One-way analysis of variance (ANOVA) was used for the comparison of each group. The p-value has been represented in each figure.

## 3. Results

### 3.1. Cell Pairing

The MFC uses a two-step protocol for the cell loading process. In the first step, THP-1 cells are loaded from Inlet A, followed by a subsequent wash step to remove excess cells. The next step continues with the loading of A549 cells from Inlet B. The washing step is repeated to flush out excess A549 cells in the flow-through channels. The process of cell trapping and pairing takes about 18 min.

### 3.2. Cell Electrofusion

**Figure 4:**
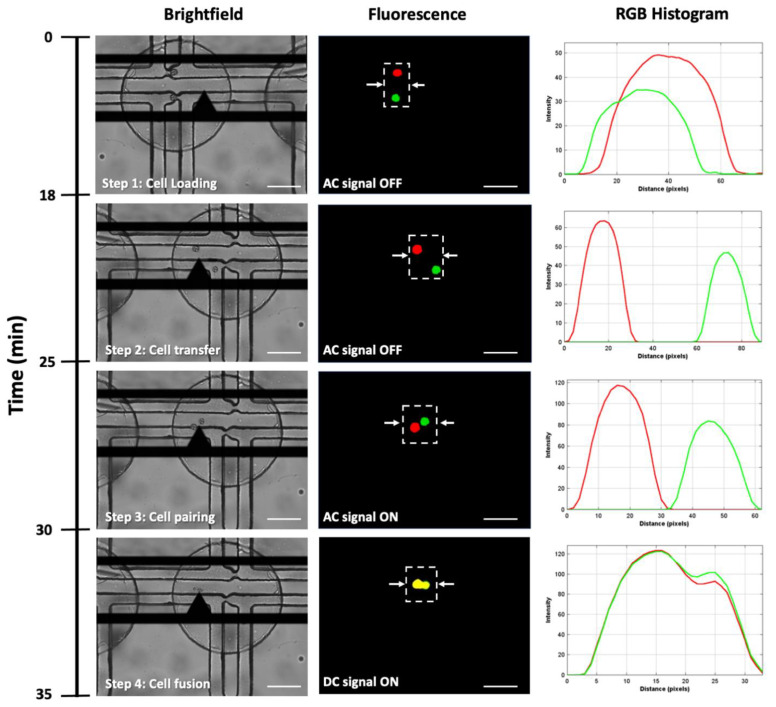
A magnified image sequence for cell electrofusion process along with brightfield image, fluorescence image, and RGB histogram. Step 1: Cell loading process with trapped THP-1 and A549 cells. Step 2: Transfer of cells into the fusion well by flipping the MFC. Step 3: Cell–cell contact is achieved by applying AC electric field. Step 4: After applying the DC pulse, the color of the fused cell changed to yellow. The RGB histogram represents the position and respective color of cells. The white arrows in the fluorescence images indicate the region across which the histogram is plotted. Scale bar: 50 µm.

The cell electrofusion process in our device was studied by analyzing fluorescence signals at different timestamps (Figure 4). The complete cell fusion undergoes various stages such as cell–cell contact, electroporation of membranes by short DC pulse, and finally, the exchange of cytosols. The electrofusion process is analyzed by observing the fluorescence dye exchange over properly paired cells using ImageJ. Fusion efficiency is defined as the total number of cells showing fluorescence exchange among properly paired cells (cell pairing consisting of one THP1 and one A549 cell). An RGB histogram for red (THP-1), green (A549) and yellow (fused cell) colors can be represented by RGB codes as (255, 0, 0), (0, 255, 0), and (255, 255, 0), respectively. At time *t* = 0, the fluorescence image shows red and green colored stained cells distinctly. After applying the DC pulse, the color of the cell changes to yellow, indicating a complete cell fusion process, as shown in Figure 4.

#### Effect of Electric Field on Fusion Efficiency

A biological cell is modeled as a particle having a conductive interior (cytoplasm) encapsulated by a thin insulating layer (lipid bilayer membrane). Equation (1) defines the membrane electric voltage ($V_{m})$ of an isolated spherical cell under the influence of an electric field ($E_{0}$).

(1) $$V_{m}=\frac{3}{2}aE_{0}cos\left(\theta \left[1-exp\left(-\tau /\tau_{m}\right)\right]\right)$$

As can be seen from the equation, the presence of the time component explains the exponential effect on the voltage generated across the plasma membrane. The angle between normal to the membrane and the electric field vector is defined as θ, and $\tau_{m}$ is the time constant of membrane charging given by

(2) $$\tau_{m}=aC_{m}\left(\frac{1}{\sigma_{cell}}+\frac{1}{\sigma_{ext}}\right)$$

where, $\sigma_{cell}$ and $\sigma_{ext}$ are the interior and exterior conductivity of the cell. The $C_{m}$ is the membrane capacitance per unit area with a typical value of 10 mF/m^2^. When the pulse duration is equal to or higher than 5∗$\tau_{m}$, the membrane gets charged entirely, and Equation (2) is reduced to Equation (3) given as

(3) $$V_{m}\left(\theta \right)=\frac{3}{2}\;aE_{ext}cos\left(\theta \right)$$

The temporary membrane perforation occurs if the value is 1V at room temperature. This critical value ($V_{c}$) is of particular interest since it helps to optimize the values of pulse strength and duration required for efficient cell electrofusion. However, if the membrane voltage is higher than the critical value, the cell membrane cannot be recovered, and there is permanent damage.

**Figure 5:**
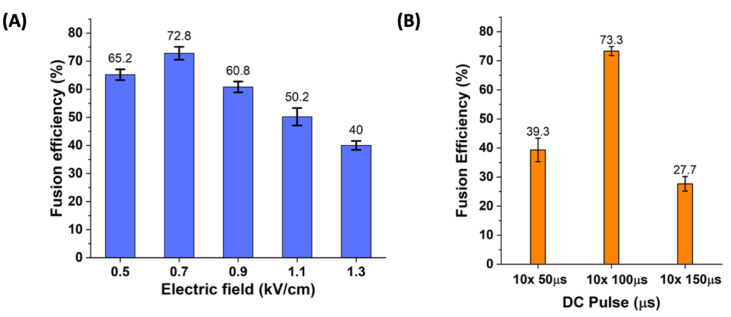
Effect of electric field on fusion efficiency (**A**) The maximum fusion efficiency of 72.8% was achieved when the electric field strength was 0.7 kV/cm. (**B**) The effect of the duration of DC pulses applied was also studied. The DC pulse duration varied from 50 µs to 150 µs. The number of pulses was fixed to 10. The highest fusion efficiency was observed when the duration of the pulse was 100 µs. Error bars, mean ± s.d., (***n*** = 3) (***p*** < 0.05).

After the washing step, the MFC was flipped and the AC signal was applied for performing the cell pairing process. We observed a pairing efficiency of around 87% (~610 cells/experiment). Next, we studied the effect of the electric field on the fusion efficiency among perfectly paired single THP-1 and single A549 cells. We observed a critical value of 0.7 kV/cm (Figure 5A), at which maximum fusion efficiency of 72.8% (~445 cells/experiment) was achieved. The fusion efficiency followed a downward curve beyond this value, with an increase in the electric field value. At an electric field less than 0.7 kV/cm, we observed incomplete cell fusion as the electric field was insufficient for the membrane perforation. As the voltage increased, we observed a decrease in the fusion efficiency with an electric field greater than 0.7 kV/cm. The decrease in the fusion efficiency was due to the incomplete cell fusion observed due to the permanent membrane perforation in which the damage is caused to the cell due to a high electric field. We also studied the effect of different duration of DC pulse on fusion efficiency, as shown in Figure 5B. We chose a range of values from 50 µs to 150 µs in three equal intervals with the number of pulses fixed to 10. The fusion efficiency was relatively lower with a 50 µs pulse duration as we observed incomplete cell fusion. We then increased the pulse duration to 150 µs and observed cell damage to a large extent due to prolonged pulse exposure. The maximum fusion efficiency was achieved when the pulse duration was 100 µs, as shown in Figure 5B. The higher time duration (greater than 100 µs) of the DC pulse resulted in permanent damage to the cells.

### 3.3. Effect of Membrane Thickness

For THP-1 and A549 cells to be collected inside the fusion well, the membrane and the channels should be precisely aligned. Several factors are also responsible for the process of cell trapping, cell retention, and cell transfer. We studied the effects of membrane thickness to optimize the operation of MFC. After fixing the height of the channel (15 µm), two sets of devices with PDMS membrane thicknesses of 12 µm and 20 µm were fabricated. The devices were tested under different flow conditions.

**Figure 6:**
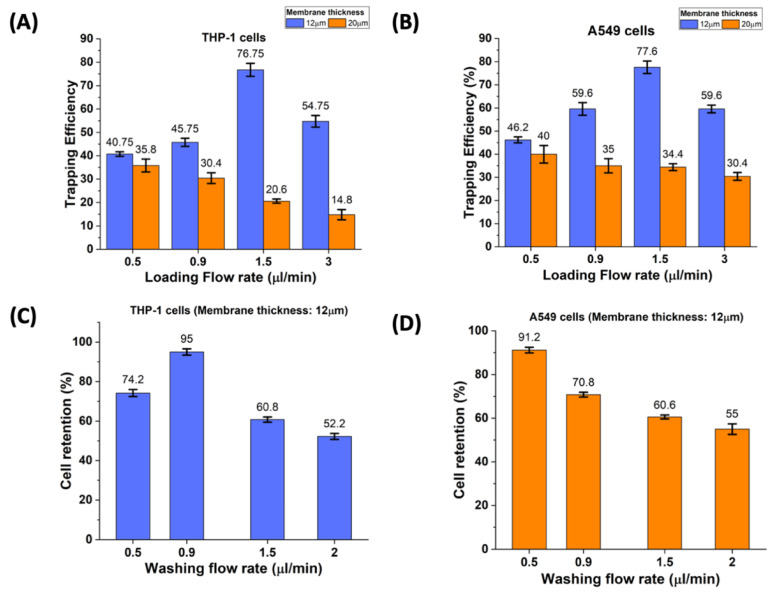
Factors responsible for THP-1 and A549 cell trapping. (**A**) Effect of cell loading flow rate on the THP-1 trapping efficiency. (**B**) Effect of cell loading flow rate on the A549 trapping efficiency (**C**,**D**) Effect of washing flow rate on cell retention of THP-1 and A549. An efficiency of 95% and 91% were achieved for THP-1 cells and A549 cells, respectively. Error bars, mean ± s.d., (***n*** = 3) (***p*** < 0.05). This optimization step was carried out on non-fluorescence labelled cells.

We observed a considerable decrease in trapping efficiency with an increase in the membrane thickness for both cell types. At higher membrane thickness, the cells could not get trapped as they slipped through the trapping area due to excessive fluid leakage (Figure 6A,B). After observations from the previous step, we fixed the value of the PDMS membrane to 12 µm. Though we observed an increasing trend of trapping efficiencies, we needed to extract exact flow rate values for maximum efficiency. The presence of a PDMS membrane makes the flow rate a parameter of consideration. We tested our device for flow rates between 0.5 µL/min and 3 µL/min. As seen from the graphs in Figure 6A,B, the membrane thickness of 20 µm leads to very poor trapping efficiencies as most of the cells were flushed away from the trapping sites. At membrane thickness of 12 µm with higher flow rates (>1.5 µL/min), cells do not get trapped at trapping sites and flow through the membrane area. However, at lower flow rates (<0.5 µL/min), we observed multiple cell trapping and some cell clogging too. An average trapping efficiency of 77% (~770 cells/experiment) was observed at a flow rate of 1.5 µL/min for both THP-1 and A549 cells.

### 3.4. Effect of Washing Flow Rate

Excess cells need to be washed away to avoid multi-cell trapping and channel clogging. The washing flow rate is another crucial parameter; if chosen, a wrong value reduces efficiency. The cell retention value was calculated based on the percentage of cells which remained at trapped sites after the washing step. During the cell loading process, THP-1 cells were first loaded, followed by A549. For the washing step, too, washing of THP-1 cells was performed first. A relatively slower washing flow rate ensured maximum cell retention at trapping sites. As seen from the graph, cell retention for THP-1 cells was 95% (~731 cells/experiment) at a flow rate of 0.9 µL/min, and that for A549 (~700 cells/experiment) was 91% at 0.5 µL/min (Figure 6C,D).

### 3.5. Characterization of Fused Cells

A fluorescence exchange within a few seconds after the application of the DC pulse was observed. A shift in color indicated that the fluorescence dye had moved to the nucleus. An AC signal was applied after complete cell fusion for the membrane reorganization. The process of cell electrofusion required approximately 35 min. The fused cells were transferred to a single well plate for further culture.

**Figure 7:**
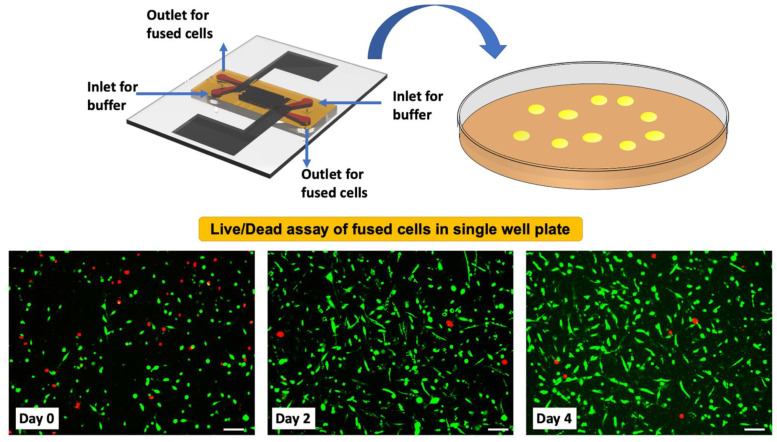
Live/dead assay. The fused cells were transferred from the chip to a single well plate for long-term cell culture. The cells were treated with a live/dead assay kit on day 0, day 2, and day 4, respectively. The high cell viability was observed even on day 4. This study was performed using non-fluorescence labelled cells. Scale bar: 100 µm.

A live/dead assay was performed to investigate the viability of cells in a single well plate. The cells were stained (LIVE/DEAD™ Cell Imaging Kit, Thermo Fisher Scientific, USA) and were observed on day 0, day 2, and day 4. As seen from Figure 7, the cell viability increased from day 0 to day 4.

We also performed an experiment by seeding the THP-1 and A549 cells together for 4 days as a negative control for the cell electrofusion experiment. We did not observe any significant fluorescence exchange over the cells indicating that negligible fusion occurred without any fusing stimuli (Figure S6).

### 3.6. Cell Viability in 96-Well Plate

In addition to the live/dead cell assay, we studied the viability of fused cells using PrestoBlue^®^ reagent. We first used 96 well plates to culture THP-1, A549, and fused cells. A similar cell density per well was seeded in 100 µL. We evaluated the cell viability of THP-1, A549, and fused cells using the PrestoBlue assay. Resazurin (λ_max.abs_ = 600 nm) in the PrestoBlue^®^ reagent, a nonfluorescent blue compound, can be reduced in live cells by metabolism to resorufin (λ_max.abs_ = 570 nm), which is red in color and highly fluorescent.

**Figure 8:**
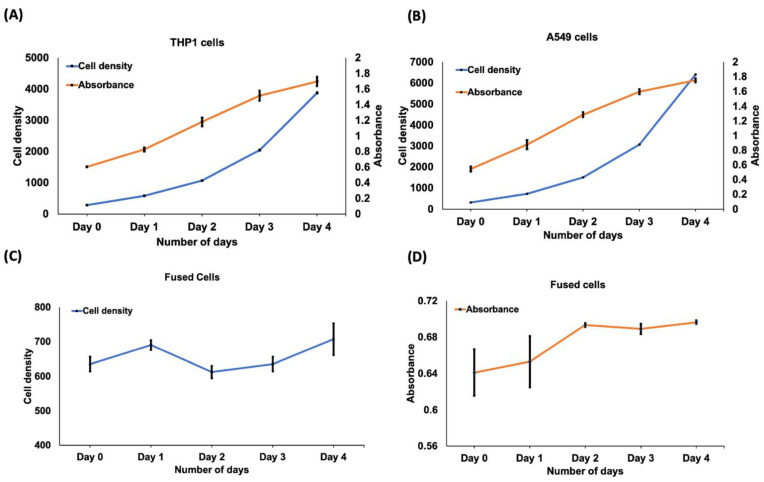
Cell viability using PrestoBlue^®^ reagent (**A**) The graph showing cell density and absorbance was plotted for THP-1 (**B**) The graph showing cell density and absorbance was plotted for A549 from day 0 to day 4. (**C**,**D**) The graph showing cell density and absorbance was plotted for fused cells from day 0 to day 4. The absorbance values indicate that the cells are viable. Error bars, mean ± s.d., (***n*** = 2) (***p*** < 0.05). This study was performed using non-fluorescence labelled cells.

Since the number of metabolically active cells proportionally correlates with the reduction level, the absorbance readings can be converted and expressed as the percentage reduction of the PrestoBlue^®^ reagent, indicating the relative cell viability. We added 10 µL of the PrestoBlue^®^ reagent to each well and incubated the plate for 2 hrs. for better readability. The absorbance was observed at 570 nm with a reference wavelength of 600 nm using an ELISA reader (BioTek 800TS) as mentioned in the datasheet. As seen from Figure 8A to Figure 8B, the absorbance shows a steady increase from day 0 to day 4 for THP-1 and A549, respectively. The steady increase in absorbance values indicates that the cells are viable. On the other hand, for fused cells (Figure 8D), the absorbance curve also followed a steady path over 4 days. The absorbance value indicates metabolically active and viable cells.

## 4. Discussion

New means of cell pairing and fusion in microfluidic systems are increasingly important since microfluidics is emerging as an important domain in cancer immunotherapy. The ability to obtain ideal conditions in a well-defined microfluidic environment for precise cell manipulation is promising for cell-based studies. We have proposed a MicroFluidic Flip-Chip combining hydrodynamic trapping and gravitational sedimentation. The cell trapping process eliminates the need for any pre-treatment, e.g., hypo-osmolar treatment. The hydrodynamic trapping process is fast and efficient, with trapping efficiencies of up to 77%. The MFC chip introduced an arrayed hydrodynamic pairing structure connected internally by a thin membrane. Although the presence of a thin membrane might look like an issue concerning leakage between the two trapping channels, a thorough experimental verification has been carried out, and it was observed that at optimum flow rates, as discussed in the previous sections, there is a negligible cross channel flow observed at lower flow rates ensuring that the cell trapping process is unaffected.

In this study, we have obtained up to 78% efficiency for pairing single THP-1 and single A549 cells, with up to 95% retention efficiencies after flipping the chip. The process of fabricating a thin through-hole PDMS membrane is a crucial step and requires precise handling. The integration of electrodes for performing electrofusion gives an edge in pairing and fusion efficiencies compared with traditional bench-top and chemical-based fusion processes. The overall fusion efficiency of 73% is achieved using the proposed MFC. High cell viability was observed even on day 4. Although the working of the chip seems to be a lengthy process, the overall time required from beginning to cell fusion takes about 35 min. This has been a considerable improvement over the traditional PEG-based fusion process. A lot of other devices have been proposed utilizing droplet microfluidics. Although, a novel technique, the efficiency is quite low at this point. A few other devices have demonstrated cell fusion with much higher efficiencies and precision. However, the devices have less throughput and their compatibility with carrying out cell fusion among heterogeneous cells has not been reported. A detailed comparison among other designs has been included in Table S1.

The proposed MFC can be designed for various cell sizes. The performance of the MFC can be improved by adjusting the diameter of the fusion well to reduce further leakage at higher flow rates. The mass-scale production of hybrids has been of more importance in recent years, apart from high viability rates. We anticipate that the proposed MFC can be scaled; however, the channel dimensions need to be carefully modified to avoid high-pressure drops across channels after arraying the trapping structures.

## 5. Conclusions

Microfluidics has enabled the precise manipulation of cells. The increasing demand for microfluidics-based devices is due to several advantages, such as a lower sample volume, ability to perform experiments at faster rates, and modification of device as per the application and type of cell. Microfluidic devices have shown immense potential for fusion application. This research aims to develop an efficient lab chip to pair single cells and carry out electrofusion efficiently. Our device design primarily focuses on two key parameters (i) single-cell trapping using passive hydrodynamics and transferring cells to fusion wells using gravitational sedimentation and (ii) performing cell electrofusion. Our hydrodynamic trapping and pairing approach is expected to cause lower cell damage due to reduced flow rates for cell trapping and lower exposure to the electric field. Our design leads us to observe the high cell viability of fused cells even after 4 days of culture.

## Data Availability

Not applicable.

## Supplementary Materials

The following are available online at https://www.mdpi.com/article/10.3390/cells10112855/s1, Figure S1. Step-by-step MFC fabrication process, Figure S2. COMSOL Multiphysics simulations of microfluidic channels, Figure S3. COMSOL Multiphysics simulations of designed electrodes, Figure S4. Step-by-step functioning of MFC, Figure S5. Effect of spin speed on PDMS thickness, Figure S6. THP-1 and A549 coculture in the absence of external stimuli, Table S1. Comparative analysis with previously reported works, Table S2. Cell trapping, pairing and efficiency calculations.
