# Supplementary material for: A Microfluidic Flip-Chip Combining Hydrodynamic Trapping and Gravitational Sedimentation for Cell Pairing and Fusion
Source: Cells. 2021 Oct 22;10(11):2855. doi: 10.3390/cells10112855 (PMC8616069; doi:10.3390/cells10112855)
Supplement: Supplementary file 1 [file cells-10-02855-s001.zip › cells-1390661-supplementary.pdf]

Supplementary Information

A microfluidic flip-chip combining hydrodynamic trapping and gravitational sedimentation for cell pairing and fusion

Gaurav Pendharkar†[1], Yen-Ta Lu†[2,3], Chia-Ming Chang[3], Meng-Ping Lu[3], Chung-Huan Lu[1], Chih-Chen Chen[1,4], and Cheng-Hsien Liu[1,4]

[1] Department of Power Mechanical Engineering, National Tsing Hua University, Hsinchu, Taiwan, R.O.C.
[2] Chest Department, MacKay Memorial Hospital, Taipei, Taiwan, R.O.C.
[3] Department of Medical Research, MacKay Memorial Hospital, New Taipei City, Taiwan, R.O.C.
[4] Institute of Nanoengineering and Microsystems, National Tsing Hua University, Hsinchu, Taiwan, R.O.C

E-mail: liuch@pme.nthu.edu.tw

† G. Pendharkar and Y. T. Lu contributed equally to this work

1. Fabrication of Microfluidic flip-chip

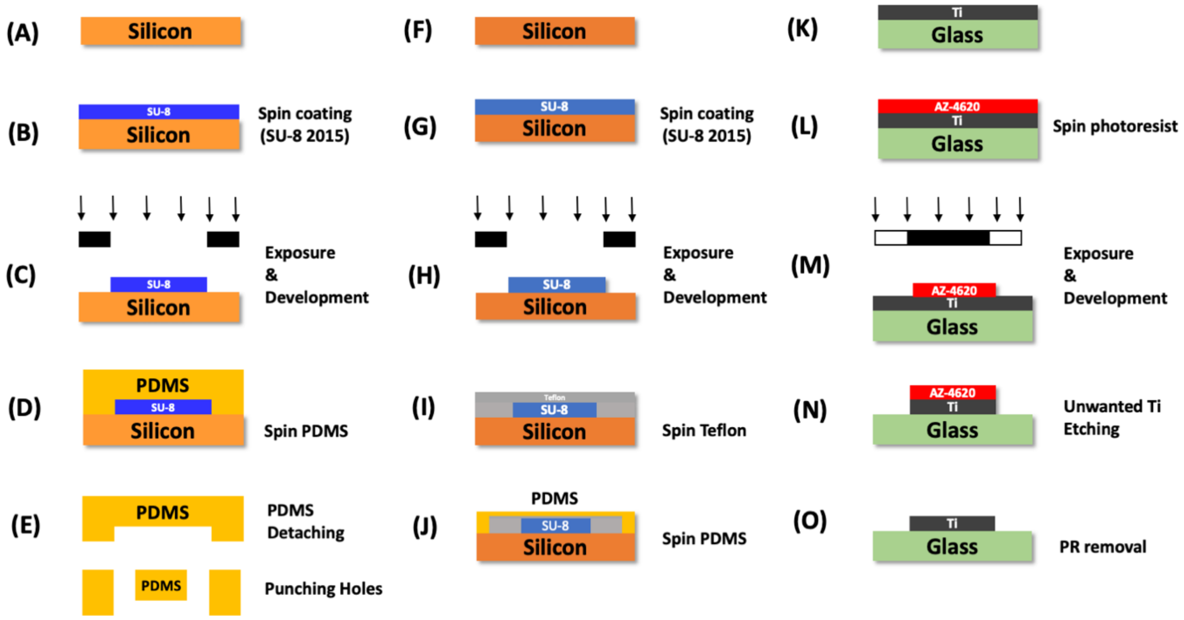

Figure S**1.** Step-by-step MFC fabrication process: (a-e) The fabrication process of PDMS mold is shown. The SU8 is UV exposed after spinning on a silicon wafer. The unwanted SU8 is removed using the developer. The PDMS mixed in 1:10 ratio is poured on SU8 master after removing bubbles and cured at 65 °C for 1.5 hrs. Finally, the holes are punched for the inlets and outlets (f-j) A thin membrane fabrication is explained here. The SU8 pillars with a height larger than the required PDMS thickness are fabricated. A thin layer of Teflon is coated on this SU8 master for easy peel-off of PDMS. The PDMS mixed with n-hexane is spin-coated on this master. (k-o). The fabrication flow explains the titanium patterning using AZ4620 photoresist. Finally, all the layers are bonded using oxygen plasma.

2. Finite element method (FEM) based simulation using COMSOL Multiphysics

Using COMSOL Multiphysics, flow field and electric field simulations were performed. Figure S.I.2-A shows a 3D simulation of the velocity profile across the microchannel. The geometry was drawn in AutoCAD and imported to COMSOL Multiphysics. The Laminar flow module was used for fluid flow simulations. For fluid flow simulations, a no-slip boundary condition was used with water as the medium. The meshing was set at physics-controlled mesh. The meshing was set to fine at microchannels and membrane area joining two channels and normal to the other areas. A stationary study was performed with solver configuration set to MUMPS. As seen from the simulation results, very little leakage is observed through the membrane. Although a cross channel flow is observed in the presence of membrane, it hardly causes any issue at lower flow rates while trapping the cells. Figure S.I.2-B shows the profile of pressure drop across the channel. Figure S.I.2-C shows the orientation of the simulated model.

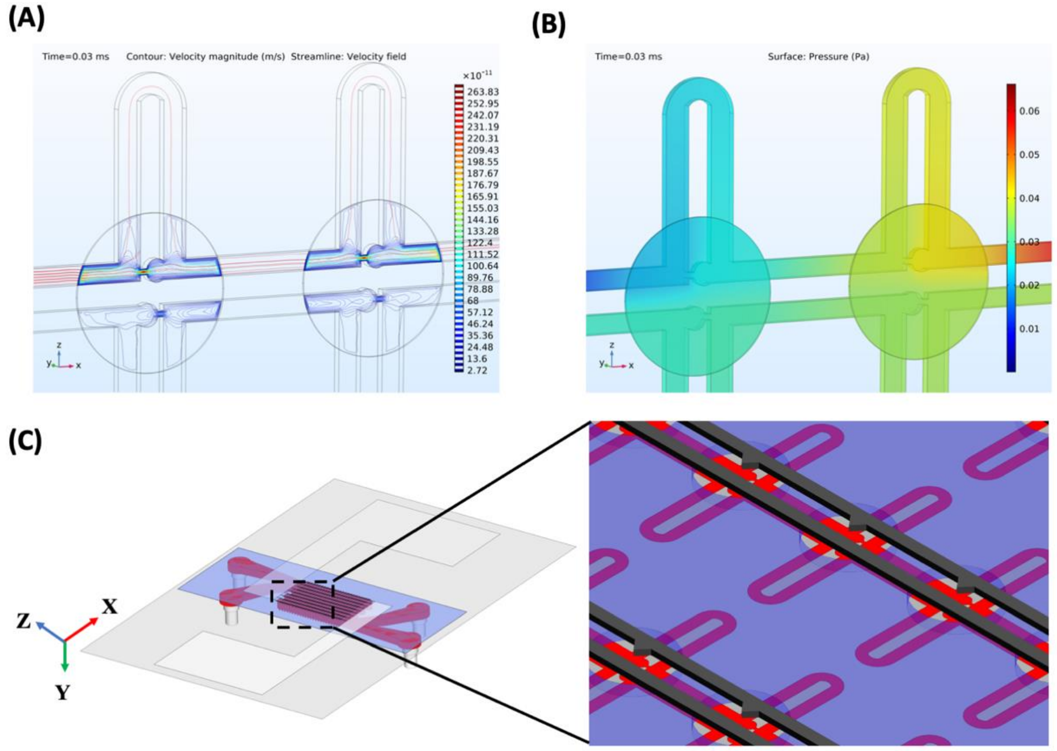

Figure S2. COMSOL Multiphysics simulations (a) Simulation of velocity magnitude in the absence of cells. The fluid velocity is higher at trapping structures than bypass channels. (b) Once the cells are trapped, the velocity near the trapped sites decreases while the velocity at bypass channels increase.

We also studied the effect of different geometries on the electric field. The electrostatics module was used for the electrode simulations. The terminal electrode was given values of 10Vpp while the other electrode was defined as the ground electrode. The meshing was done using physics-controlled mesh. A stationary study was performed with MUMPS. The Dielectrophoresis phenomenon occurs when there is a non-uniform electric field generated between the two electrodes. We designed two different shapes of electrodes, rectangular and triangular, and did a finite element method simulation to its effect on the generation of the gradient of the electric field. We observed that the highest gradient was at the tip of the electrode. As can be seen in rectangular electrodes (Figure S.I.3-A), rectangular electrodes have two endpoints, and thus the field is distributed. However, for triangular electrodes, the electric field concentration is much higher at the tip. The illustration in Figures S.I.3-A and Figure S.I.3-B shows the effect of the electric field on cell trapping. In triangular electrodes, the cells are much closer to each other, which is the most suitable condition for cell electrofusion.

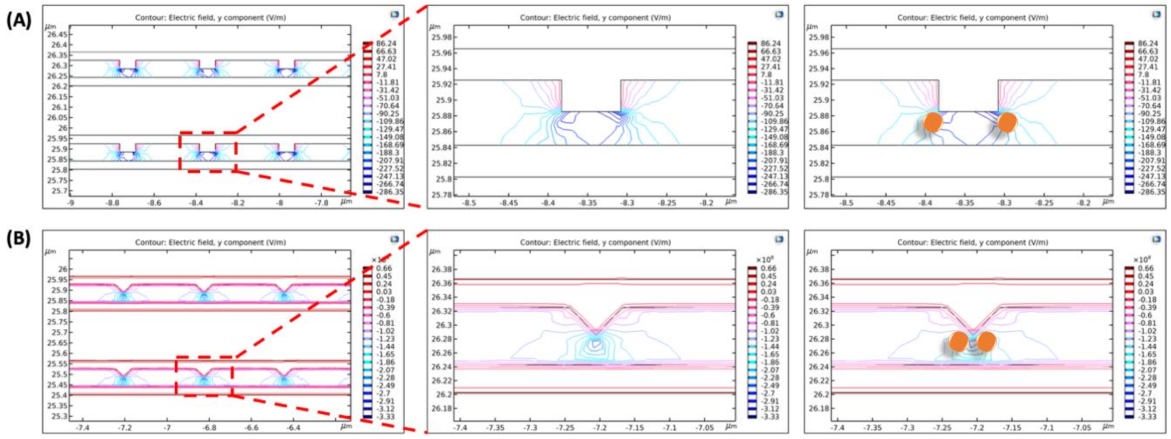

Figure S3. COMSOL Multiphysics simulations (A) Electric field simulation for rectangular electrodes. The adjacent figure shows a closer view of the electric field near the electrode. The last figure shows an illustration of the trapping of cells in the presence of an electric field. (B) Electric field simulation for triangular electrodes. The adjacent figure shows a closer view of the electric field near the electrode. The last figure shows an illustration of the trapping of cells in the presence of an electric field. The presence of gradient focused at the tip of triangular electrodes helps towards a closer cell-cell contact than rectangular electrodes. The presence of gradient is observed at the tips present at the extreme corners of the intersection of length and width sides.

3. Step-by-step functioning of MFC

Cell loading of THP-1 was accomplished by infusing 1ml of cell suspension in a fusion buffer from "Inlet A" and drawing the cells through the device at 1.5µl/min using a syringe pump. After most of the trapping sites were filled with cells, a cleaning step was performed. A fusion buffer was flushed into the channels at a little reduced flow rate (0.5µl/min). This process ensured the removal of excess cells while maintaining the already filled trapping sites intact. Next, the second type of cells, i.e., A549, was introduced from "Inlet B" with a flow rate of 1.5µl/min. The syringe pump was kept on until most of the sites were filled with cells. A washing step was performed to remove excess cells by replacing cell suspension with a fusion buffer. No significant effect concerning cell unloading from the traps was observed during the second step of cell loading (Figure S.I.4, Step 1).

At last, the tubing was removed from the chip, and the holes were plugged. The MFC was flipped gently in which the trapping sites are now on the channel's ceiling, and the fusion-wells are on the floor (Figure S.I.4, Step 2), allowing the captured cells to fall off from the trapping sites to the fusion wells by gravity. After the cells were settled in the fusion well, the uncaptured cells were washed away from the device by slowly injecting fusion buffer. The cell trapping and transferring procedure take approximately 15-18 minutes.

After the cell trapping process is complete, the chip is flipped, and all the cells are transferred to the fusion well. An alignment signal (V_{PP}: 10V, frequency: 1MHz) was applied between the electrode array present at the bottom (Figure S.I.4, Step 3). A low conductivity buffer solution is necessary for the dielectrophoresis phenomenon to occur. Alignment signal induces positive DEP force on the cells aligning them as pairs with high efficiency. In the next step, a DC pulse (Duration: 100µs, Number of pulses: 10) was applied to induce temporary cell membrane perforation (Figure S.I.4, Step 4). The optimum value of the DC field helps in cell membrane reconstruction because of the cell's self-recovery ability and resealing of the cell membrane after the cytoplasm exchange of the paired cells by maintaining cell viability. The chip was later flipped (Figure S.I.4, Step 5). The fused cells can be removed by pumping buffer solution through Outlet A and Inlet B, and the cells are collected from Inlet A and Outlet B (Figure S.I.4, Step 6).

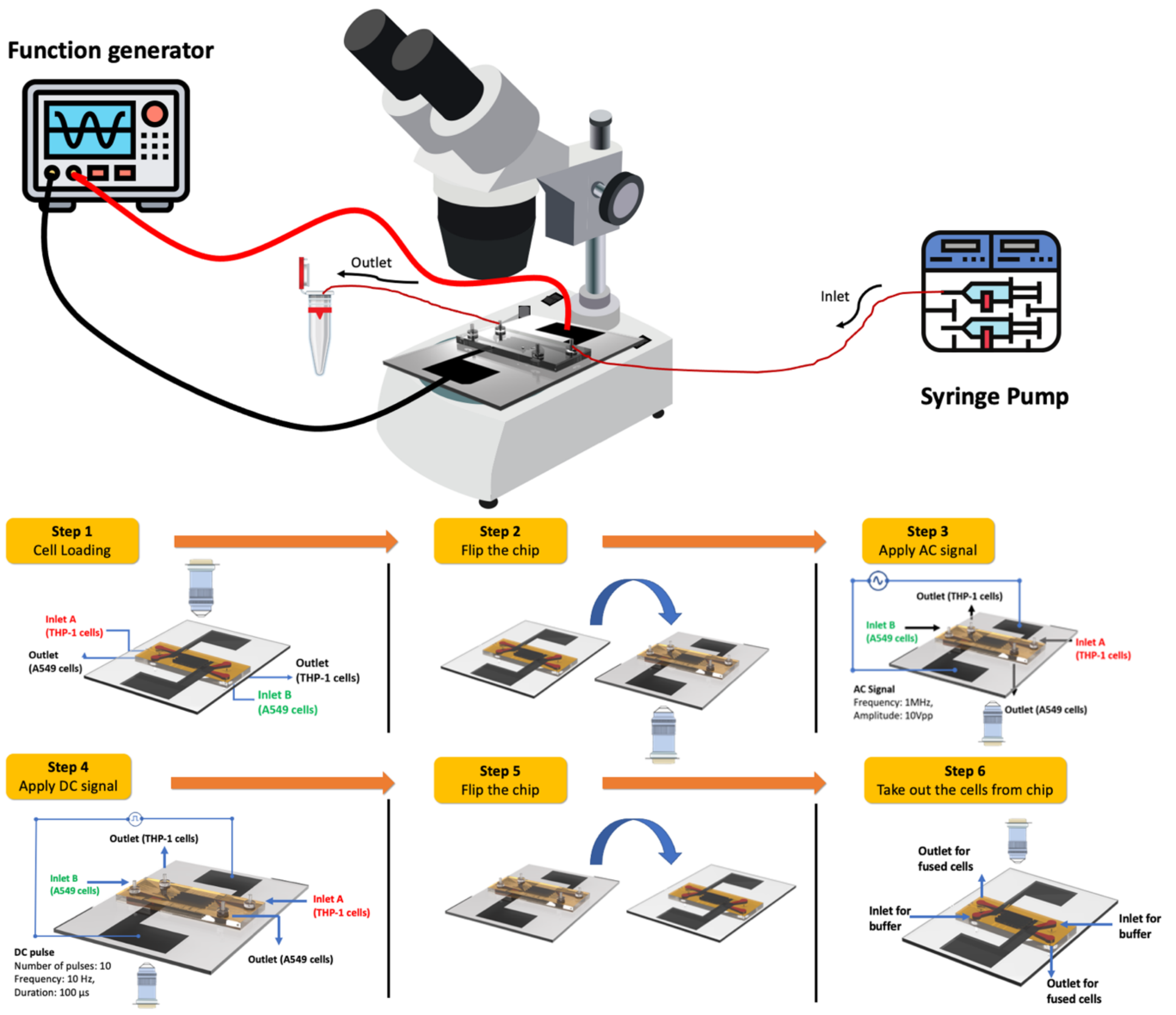

Figure S4. Step by step cell loading protocol. The cells can be loaded serially or in parallel. The illustration explains the serial loading of cells. (Step 1) THP-1 cells are loaded from Inlet A. Once the cells occupy all the trapping sites, a washing step is performed. A549 cells are loaded from Inlet B. Once all the sites are occupied by cells, a washing step is performed to ensure no excess cells present in the chip. (Step 2) Flip the chip to transfer all the cells to the microwells. (Step 3) In this step, the cell-cell contact is achieved by applying the AC signal (Frequency: 1Mhz, Amplitude: $10V_{PP}$). (Step 4) Apply DC pulse for carrying out cell fusion (Number of pulses: 10, Duration: 100 µs) (Step 5) Flip the chip. (Step 6) Flow buffer from the two inlets and collect fused cells. The microscopic lens shows the direction from which the images were recorded, as a combination of upright and inverted microscopes was used.

4. Effect of spin speed vs. PDMS thickness

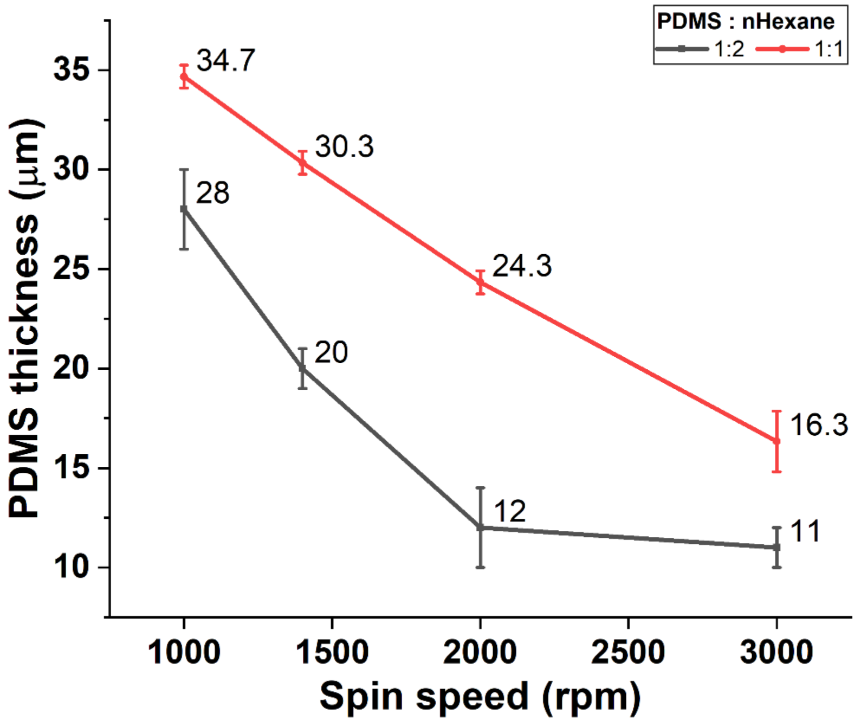

Figure S5. Effect of spin speed on PDMS thickness. As shown from the graph, we performed the analysis by varying PDMS: n-hexane dilution ratio. For both the dilution ratios, we observed that as spin speed increases, PDMS thickness decreases. The experiments were conducted in quadruplet or triplet, and data are presented as mean ± standard deviation

5. THP-1 and A549 coculture in the absence of external stimuli

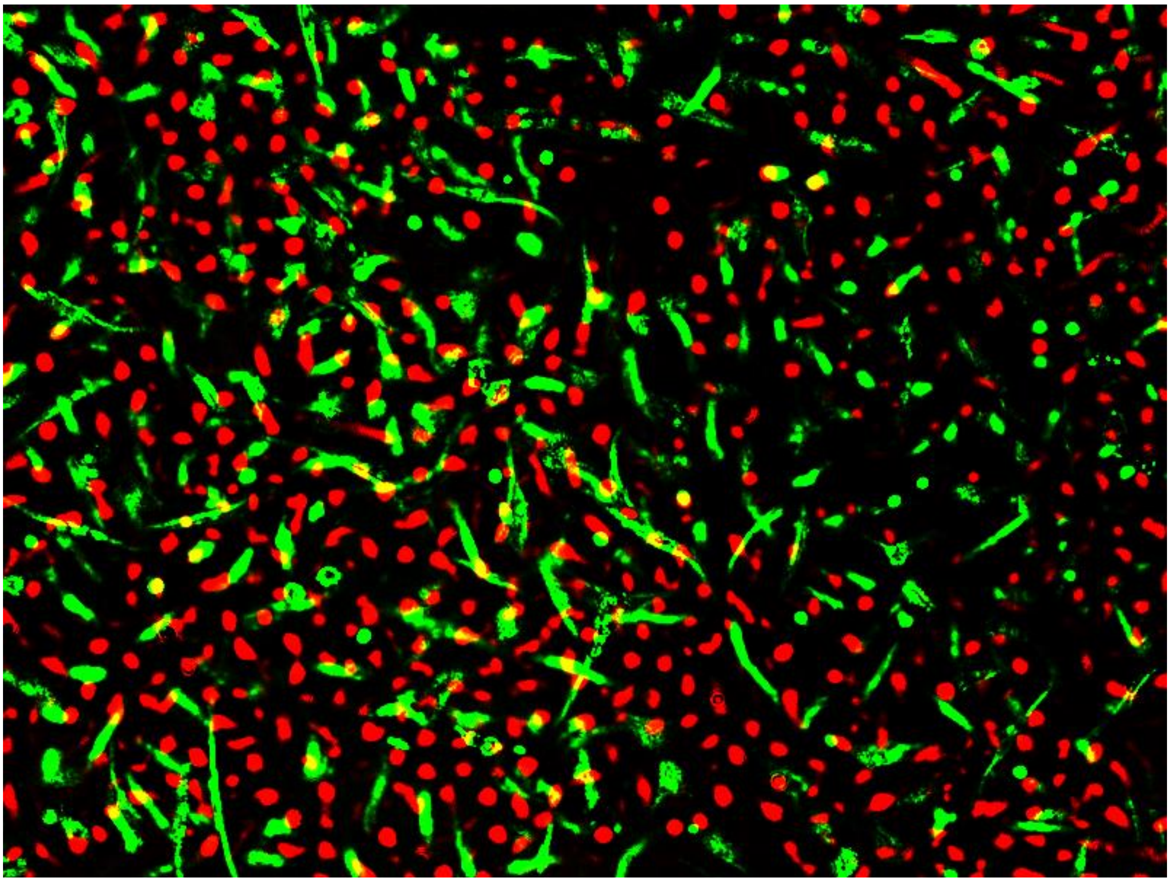

Figure S6. We performed an experiment involving coculture of THP1 cells (Red-colored, labeled by CMTPX) and A549 cells (Green colored, labeled by CMFDA). The cells were cultured for 4 days in the absence of any external stimulus responsible for fusion. We observed no doubly labeled cells indicating very negligible fusion in the absence of an external stimulus.

Table S1: Comparative analysis with previously reported works

		Cells type	Throughput	Mechanism	Fusion electrical parameters	Fusion Efficiency (FE)/Pairing Efficiency (PE)	Fabrication Complexity	Approximate Area per cell pair	Remark	Cell Viability Data
[1]	Skelly et.al., Nat. Methods (2009)	3T3-3T3, mESC-mEF (Heterogeneous)	6000	Fusion between electrodes	1000V and Hypoosmolar pretreatment	FE = 50% Mem. reorg eff = 89%	Low	30x15 μm^2	High electric field and pretreatment required	✓
[2]	Gel et.al., Biomicrofluidics (2010)	L90 (Homogeneous)	400	Fusion between electrodes	DC: 4 V, 300 µs	FE = 95%	Medium	30x30 μm^2	Cell fusion is only among homogeneous cells	✗
[3]	Kemna et.al., Electrophoresis (2011)	B-cell and NS-1 (Heterogeneous)	783	Fusion between electrodes	2.5kV/cm, 6 x 100µs Pulse	51%	Low	8x50 μm^2	Low throughput	✓
[4]	Kimura et.al., Electrophoresis (2011)	Jurkat, L929, K562, HL60	6000	Fusion between vertical electrodes	10 V, 50 ms	70%	Low	25x8 μm^2	The membrane is made using proprietary material. Difficulty in removing fused cells	✗
[5]	Sen et.al., Lab on a chip (2013)	3T3 and ES (900) (Heterogeneous)	900	Fusion between electrodes	AC: 1Mhz $8V_{pp}$	44%	Low	25x10 μm^2	Low throughput and fusion efficiency. Chip demonstrated	✗

									only for cell pairing	
[6]	Wada et.al., Biotechnology and Bioengineering (2014)	3T3 (Homogeneous)	100	Fusion between electrodes	———	14%	Low	500x28 μm²	Low throughput and fusion efficiency. Chip is focused for avoiding nuclear mixing	✗
[7]	Dura et.al., Lab on a chip (2014)	3T3 and BA/F3 (Heterogeneous)	6000	Fusion between electrodes	1000V and Hypoosmolar pretreatment	FE = 86%	Low	45x15 μm²	Pretreatment of cells required	✗
[8]	Hu et.al., Lab on a chip (2015)	Her2 (Homogeneous)	824 droplets	Cell pairing	———	Pairing Eff. = 75%	Low	Droplet size = 100 μm	Chip is a good alternative for cell pairing. However, cell fusion has not been demonstrated	✗
[9]	Yang et.al., Nat. Scientific Report (2016)	HeLa and A549 (Heterogeneous)	783	Optically induced cell fusion	AC: 12 to 8kHz @ 0.72V	50%	High	30x30 μm²	No data about cell viability	✗
[10]	Wu et.al., Lab on a chip (2017)	HeLa (Homogeneous)	3264	Cell pairing through IDT	AC 4MHz $12V_{PP}$	74.2%	Medium	Diameter of trapping site = 160 μm	Only cell pairing is demonstrated.	✗
[11]	Hsiao et.al., Biomicrofluidics (2018)	Pan 1 and A549 (Heterogeneous)	106	Optical fusion	DC: 10 V	9.67%	Medium	30x40 μm²	Less fusion yield and no viability data	✗
[12]	Schoeman et.al., Nat. Scientific Reports (2018)	HL60 (Homogeneous)	500	Fusion inside droplet	DC: 3V	5%	High	Droplet volume = 18pL	Homogeneous cells and lower fusion efficiency.	✗

									Also, lack of viability data	
[13]	Huang et.al., Lab on a chip (2018)	HeLa–C2C12	6000	PEG based cell fusion	– –	15%	Low	20x40 μm²	PEG based. Low fusion efficiency	✗
[14]	Zhu et.al., Sensors and Actuators B (2019)	HUVEC, HeLa and MCF-7 (Heterogeneous)	80	Cell pairing	No Electric field	93%	Low	40x50 μm²	It can be used only for cell pairing	✗
[15]	He et.al., Biomicrofluidics (2019)	HeLa (Homogeneous)	864	Cell fusion between electrodes	AC: 1MHz 9 Vpp DC: 10V, 40ms, No. Of Pulses = 5	26%	Low	55x25 μm²	Low throughput	✗
[16]	Chen et.al., Cell Reports, (2019)	BCC and MSC (Heterogeneous)	3000	Cell pairing	– –	20%	Low	100x100 μm²	Although a great hydrodynamic technique, the pairing efficiency is low in its current form	✗
[17]	Li et.al. Analytical Chemistry (2019)	HeLa and dHL-60	400	Cell pairing	– –	20%	Low	~ 30x20 μm²	Good for cell pairing. However, no cell fusion is demonstrated	✗
[18]	Our Work	THP1 and A549 (Heterogeneous)	1000	Cell fusion between electrodes	AC 1MHz $10V_{PP}$ DC 20V	71%	Medium	~ 40x40 μm²	Larger chip area	✓

Notes:

$$\text{Trapping efficiency} = \frac{Number\ of\ single\ cell\ trapping\ sites}{Total\ number\ of\ trapping\ sites} \times 100$$

$$\text{Fusion efficiency} = \frac{Number\ of\ perfectly\ fused\ cells}{Total\ number\ of\ sites\ with\ one\ on\ one\ pairing} \times 100$$

Table S2. Cell trapping, pairing and efficiency calculations

Total pairing structures in MFC	**1000**	
	**THP-1 (1000)**	**A549 (1000)**
Cell trapping efficiency	(~ 77%) = 1000*77%= 770	(~ 77%) = 1000*77%= 770
Cell retention efficiency	(% 95%) = 770*95% = ~ 731	(~ 91%) = 770*91% = ~ 700
Cell pairing efficiency (single THP-1 and single A549)	(~ 87%) = 610	
Fusion efficiency among perfectly paired cells	(~ 72.8%) = 445	
